# Enabling transparent research evaluation: A method for historical RCR retrieval using public NIH metadata

**DOI:** 10.1371/journal.pone.0340697

**Published:** 2026-01-20

**Authors:** Fabian Haupt, Jan F. Senge, Hendrik von Tengg-Kobligk, Wolfram A. Bosbach

**Affiliations:** 1 Department of Diagnostic, Interventional and Pediatric Radiology, Inselspital, Bern University Hospital, University of Bern, Bern, Switzerland; 2 Department of Mathematics and Computer Science, University of Bremen, Bremen, Germany; 3 Dioscuri Centre for Topological Data Analysis, Warsaw, Poland; 4 Department of Nuclear Medicine, Inselspital, Bern University Hospital, University of Bern, Switzerland; Al Muthanna University, IRAQ

## Abstract

The demand for improvement of research output evaluation by the international science community has been recently formulated as the Declaration on Research Assessment (DORA) by the American Society for Cell Biology. The Relative Citation Ratio (RCR), introduced by the National Institutes of Health (NIH) is a novel metric indicating the influence of a publication on its peer group — publications from the same research field, as determined by co-citation analysis. The RCR can be viewed for the actual month. While historical RCR data exists within the NIH database, it is not exposed in a way that allows direct or simple access for researchers. We present a method to reconstruct otherwise inaccessible RCR data. A Python-based approach was deployed to extract RCR data from the NIH database and to plot RCR-values for every time point since introduction of the database. This method demonstrates the feasibility of recovering historical bibliometric information and may contribute to more transparent and accountable use of metrics in academic evaluation — in line with the goals of DORA and open science initiatives.

## Introduction

The Relative Citation Ratio (RCR), introduced by the National Institutes of Health (NIH), was developed to address key limitations of traditional citation-based metrics by providing a field-normalized, article-level measure of influence [1,2]. The RCR is defined as the total number of citations divided by the yearly number of citations of publications in the same field — defined by the NIH. It is article specific and only accounts for PubMed-listed sources [1]. As such, the RCR serves as a field-normalized measure of individual article influence — making it increasingly relevant in academic evaluation and funding decisions. An RCR above 1.0 reflects citation rates above the median of PubMed-listed publications of the same scientific area.

In addition to the RCR, the h-index remains one of the most commonly applied bibliometric indicators in academic evaluation. It is defined as the maximum value h such that a researcher has published h articles that have each been cited at least h times. This composite measure is intended to capture both productivity (number of publications) and citation impact (number of citations), and therefore continues to be frequently used in promotion and funding contexts [3].

The Declaration on Research Assessment (DORA), developed in 2012 by the American Society for Cell Biology, recognized the need for improvement of research output evaluation (sfdora.org) [4].

In Switzerland, DORA has already been signed by 60 organizations (status of December 2024). These include various foundations, such as the Swiss National Science Foundation (SNF), as well as numerous universities.

Quantitative metrics play a central role in academic decision-making, yet concerns have been raised about their transparency, fairness, and reproducibility — as highlighted by initiatives such as DORA. The University of Bern has adopted DORA in the Faculty of Medicine and included the RCR 2018 in the regulations for habilitation [5]: To successfully achieve habilitation, a candidate must — among other items — provide at least two publications with an RCR of 1 or above [6].

The common approach to obtain the RCR from the National Institutes of Health (NIH) is the utilization of iCite, https://icite.od.nih.gov/ an online tool provided directly by the NIH [7]. However, iCite provides only the current RCR, with no access to historical values — creating a gap for those seeking to verify past performance thresholds or assess citation dynamics over time.

Thus, a habilitation candidate could meet the RCR criterion but not provide the necessary proof. To address this transparency gap, we present an open and reproducible method to extract historical RCR data from NIH archives — supporting traceable, data-driven research assessment. The underlying code has been written under assistance of the large language model (LLM) ChatGPT (Chat Generative Pre-trained Transformer) by OpenAI (San Francisco, CA, USA), a language model-based bot [8].

The method presented is non-commercial, simple to apply and intended to provide researchers access to historical RCR data.

## Materials and methods

### Study design

Retrospective analysis of a database provided by the NIH.

### Ethics

No human health related data or animal experiments were involved. The data repository/ structure/ storage and handling are explained below.

### Local hardware

This study used an Intel Core i5-6200U central processing unit (CPU) at 2.30 GHz with 8.00 GB random access memory (Intel Corporation, Santa Clara, CA, USA).

### Accessing data storage

The database for the actual month was accessed through the online interface of iCite (https://icite.od.nih.gov/analysis, date of access October 13th 2023) (6).

For historic RCR-values, databases were obtained from the NIH figshare deposit (https://doi.org/10.35092/yhjc.c.4586573) (13), which is updated on a regular basis by the NIH. Each month three files are deposited:

- Metadata twice (as.zip and as.tar.gz),
- as well as the open citation collection.

The relevant file containing the data for accessing historic RCR-values is the metadata.

### Data structure

Each metadata file is for its respective date a complete register of all PubMed-listed manuscripts, sorted by the PubMed ID (PMID) including benchmark information for each individual manuscript, such as RCR, total citation count, or year of publication.

### Data download and storage

The metadata file name (icite_metadata.zip) does not include any identifier for the respective date, therefore each file was stored in a folder system with one directory per month. The folders were named after their respective time point in the format YYYYMM.

### Code generation

Due to the size of the databases contained in the metadata files, a computer code was written for data extraction. The code applied for RCR-value extraction was written in Python language (Python Software Foundation, DE, USA) by W.A.B. Running of the code was performed in Spyder (v5.4.3, Anaconda, TX, USA). Metadata from 10/2022–07/2023 was analyzed. All code and data handling steps were designed to support reproducibility and align with open science principles. Code debugging was supported by the LLM [8]. It has demonstrated in the past a cetrain level of competence in various medicine related tasks [9].

### RCR-value extraction/ *code-1.py*

Running code-1.py starts a search in the monthly databases from top to bottom for the specified PMID. A local copy is saved for each month of the database entry. Searching one metadata file for one PMID required in this study approximately 20 minutes.

S1 File supplementary example code of code-1 (Publication A).

S3 File: extracted data entries (Publication A, and B)

### Data summary/ *code-2.py*

Running code-2.py allows data aggregation and the making of Table 1 and Fig 2.

**Table 1:** RCR and citation count, retrieved from the iCite databases.

Publication A (PMID 31278348)			Publication B (PMID 34067405)		
Date	Relative Citation Ratio	Citation Count	Date	Relative Citation Ratio	Citation Count
202006	N/A	2			
202007	N/A	2			
202008	N/A	2			
202009	0.86	2			
202010	0.72	2			
202011	0.7	2			
202012	0.7	2			
202101	0.49	2			
202102	0.49	2			
202103	0.61	3			
202104	0.58	4			
202105	0.69	5			
202106	0.77	6			
202107	0.9	7			
202108	0.86	7			
202109	0.86	7			
202110	0.79	7			
202111	0.76	7			
202112	0.73	7			
202201	0.73	7			
202202	0.68	7			
202203	0.66	7			
202204	0.63	7			
202205	0.62	7			
202206	0.6	7			
202207	0.66	8			
202208	0.65	8			
202209	0.63	8			
202210	0.68	9	202210	1.2	2
202211	0.66	9	202211	1.11	2
202212	0.64	9	202212	1.03	2
202301	0.63	9	202301	0.96	2
202302	0.69	10	202302	0.89	2
202303	0.67	10	202303	0.82	2
202304	0.73	11	202304	0.79	2
202305	0.71	11	202305	0.72	2
202306	0.69	11	202306	0.68	2
202307	0.68	11	202307	0.65	2

Legend: N/A not available.

S2 File supplementary example code of code-2 (Publication A, and for Publication B).

### ChatGPT coding help and *README* file

As part of the open access supplement, a file named read.me is included with detailed instructions for replicating the published code. Based on the provided example of PMID 34067405, code variations can be created and run for other (e.g., any) PMID. For debugging of code-1.py and code-2.py, ChatGPT 3.5 was employed.

### Historic RCR-value extraction

The previously described steps were applied on two publications of our group: Publication A (PMID 31278348) Haupt et. al. from 2019 [10] with a maximum RCR of 0.9 in 07/2021, and Publication B Bosbach et. al. (PMID 34067405) [11] with a maximum RCR of 1.2 in 10/2022. Historic RCR-values were obtained in table format (*.csv).

## Results

### Feasibility of historic RCR-value extraction

Historic RCR-values of two sample publications, Publication A (PMID 31278348) from the field of Cellular Biology and Publication B (PMID 34067405) from the field of Public Health Sciences, were successfully retrieved using the approach outlined in the Methods section. These values, which are not directly accessible through NIH’s iCite tool, were reconstructed by systematically parsing archived NIH metadata files. The extracted data spans from the publication dates to July 2023 and is presented in Table 1.

### RCR data over time

**Fig 1:**
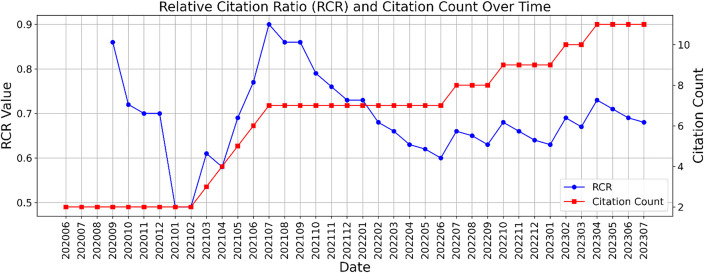
RCR-values and citation count of Table 1 for publication A plotted by Python over time, reconstructed from NIH metadata using Python.

For publication A, the RCR started from 0.86 in September 2020 at two citations with an all-time maximum at 0.90 in July 2021 with a total count of 7 PubMed-listed citations at that time point. The RCR declined after this time point, receiving 4 more citations till 2023−07. The course of the number of citations and the RCR-value over time are displayed in Fig 1.

From June 2020 on, a “saw-tooth profile” was observed. The RCR climbed multiple times whenever an additional citation was found and, dropped again in the following months where no new citations were found but peer group citations increased.

**Fig 2:**
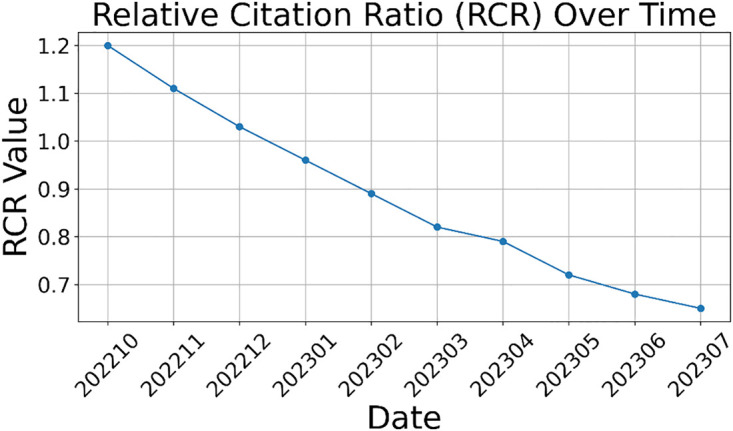
RCR-values of Table 1 for publication B plotted by Python over time.

For publication B, the RCR started in Oct 2021 above the 50^th^ percentile with a value of 1.2 from two PubMed-listed citations. The publication was not cited after this time point and the RCR continuously dropped. The course of the RCR-value over time of publication B is displayed in Fig 2.

## Discussion

### Relevance of the RCR

The h-index remains the most established bibliometric indicator for academic evaluation, combining productivity and citation impact into a single number. However, it has well-recognized limitations: it favors senior researchers over early-career scientists, varies substantially across disciplines, and is insensitive to highly cited individual publications. In contrast, the RCR provides an article-level, field-normalized measure of influence, thereby enabling more equitable comparisons across researchers at different career stages and in different domains. This conceptual distinction has led to increasing interest in RCR as a complementary or alternative metric to the h-index in promotion and funding contexts [1].

### Historical RCR extraction from NIH database

This project demonstrates the feasibility of extracting historical Relative Citation Ratio (RCR) values from publicly available NIH metadata. By providing a transparent, script-based approach to reconstruct monthly RCR values, this method addresses a significant gap in the bibliometric infrastructure: the absence of accessible historical citation influence data. While the NIH’s iCite tool provides a valuable overview of RCR at a single time point, it does not support longitudinal analysis or retrospective benchmarking [1,7]. Our contribution offers a reproducible solution that aligns with the goals of responsible metrics frameworks such as DORA and the Leiden Manifesto [4,12].

### Practical importance

The practical need for such retrospective data is evident in academic evaluation and research policy. RCR has increasingly been used to assess individual academic performance in a field-normalized manner. For example, in ophthalmology, Patel et al. showed that mean and weighted RCR better reflect individual impact than the h-index, particularly for early-career researchers [2]. Similar applications exist in plastic surgery, neurosurgery, and orthopedic hand surgery [13–15]. Our method can complement these efforts by enabling time-resolved evaluations — such as whether a publication surpassed an RCR threshold during a candidate’s qualifying period for promotion or grant eligibility.

The RCR has also been applied by funding agencies such as the NIH to evaluate research impact at the portfolio level. In program evaluations, RCR aggregates have been used to assess whether certain grant mechanisms or funding strategies correlate with greater scientific influence [16]. The availability of historical RCR data may enable novel retrospective analyses, including tracking time-based citation influence or comparing the performance of funding programs over distinct periods.

### Limitations to the presented process

From a technical perspective, our method leverages publicly available resources provided by the NIH, including monthly iCite snapshots published via Figshare [17]. While real-time RCR values can be accessed via the iCite API, historical data reconstruction is not natively supported. Our code provides a missing link by automating access to and aggregation of these data over time. Existing tools such as the R package iCiteR enable programmatic access to RCR, but do not reconstruct RCR trajectories — a gap our system addresses.

Further, integration of RCR into widely used platforms like Dimensions and ReadCube has expanded its visibility and utility [18]. Our method enhances this ecosystem by allowing institutions to analyze not just current RCR values, but also their historical progression. This enables applications in academic dashboards, evaluation audits, and institutional meta-research.

The main limitation of our method lies in its manual steps — downloading, storing, and processing monthly metadata files. While functional, these steps could be further streamlined via automation or integration into platforms like iCite. We encourage the NIH and bibliometric tool developers to explore this as a valuable feature extension. In terms of scalability, the process is primarily limited by local hardware performance and internet bandwidth. Since data retrieval relies on stable NIH figshare repositories, error margins are negligible, and adapted Python code can be executed on standard computing environments. What has been demonstrated for individual publications can, in principle, be expanded to the research output of individual scholars, research groups, or institutions, supporting large-scale bibliometric analyses.

### Limitations to the explanatory power of RCR

It is important to note that RCR, while valuable, has its limitations. It only includes PubMed-indexed publications and therefore may underrepresent influence in disciplines not well covered by PubMed indexing. At the same time, in biomedical fields where PubMed coverage is comprehensive, this focus can be seen as an advantage, since it benchmarks against the corpus most relevant for academic success in these domains. Additionally, RCR depends on co-citation networks, which may not form reliably in newly emerging or interdisciplinary fields [1]. Beyond this, the metric is bounded by contemporary citation practices — it reflects what is deemed “worth citing” at the time, which may shift over years and across disciplines.

Comparison groups underlying RCR calculations are not retrievable by users form the NIH data, as the NIH does not disclose the precise peer set used for normalization. This lack of transparency contradicts reproducibility as a major scientific requirement and limits independent verification of the metric’s validity.

Another important limitation concerns the time lag with which RCR values are provided by the NIH. Since the metric depends on accumulating co-citation networks and benchmark citation rates, newly published articles only receive an RCR after a delay of several months. This latency reduces the suitability of RCR for assessment, particularly in fast-moving research areas. Research bodies should therefore consider for which time windows alternative indicators (e.g., raw citation counts, field-normalized citation scores from other sources) or carefully defined ad hoc values may provide a more appropriate proxy until RCR estimates become available.

Furthermore, while historical RCR values can illuminate citation trajectories, they should not be misapplied for retrospective evaluation in ways that contradict DORA’s principles, since such use risks reinforcing metric-driven assessments rather than contextualized evaluation. Therefore, RCR should be used as one of several indicators and interpreted in context — as recommended by both DORA and the Leiden Manifesto [4,12].

## Conclusions

In conclusion, our method provides a practical and reproducible solution to retrieve historical RCR values. This supports more transparent, time-aware, and traceable use of citation metrics in both academic evaluation and funding contexts. By bridging this data gap, our work contributes to the broader movement toward responsible, open, and reproducible research assessment. At the same time, the inherent limitations of RCR — including its restriction to PubMed sources and the non-disclosure of comparison groups by the NIH — highlight challenges to reproducibility that must be considered when applying the metric.

## Supporting information

S1File Python code-1 (Publication A). (PY)

S2 File Python code-2 (Publication A, and B). (PY)

S3 File Extracted data entries (Publication A, and B) The study’s raw data is deposited for open access on figshare under the DOI: https://doi.org/10.6084/m9.figshare.30067852.
(PY)
