# Correction: Enabling transparent research evaluation: A method for historical RCR retrieval using public NIH metadata

**DOI:** 10.1371/journal.pone.0345661

**Published:** 2026-03-23

**Authors:** Fabian Haupt, Jan F. Senge, Hendrik von Tengg-Kobligk, Wolfram A. Bosbach

The Data Availability statement for this article is incorrect. The correct statement is: The study’s raw data is deposited for open access on figshare under the DOI: https://doi.org/10.6084/m9.figshare.30067852.
